# Fluorination modulation theory for vacuum-ultraviolet nonlinear optical crystals (I): foundation and framework

**DOI:** 10.1093/nsr/nwag259

**Published:** 2026-05-18

**Authors:** Zhihua Yang, Abudukadi Tudi, Min Zhang, Miriding Mutailipu, Fangfang Zhang, Shilie Pan

**Affiliations:** Research Center for Crystal Materials, CAS Key Laboratory of Functional Materials and Devices for Special Environmental Conditions, Xinjiang Key Laboratory of Functional Crystal Materials, Xinjiang Technical Institute of Physics and Chemistry, Chinese Academy of Sciences, China; Center of Materials Science and Optoelectronics Engineering, University of Chinese Academy of Sciences, China; Research Center for Crystal Materials, CAS Key Laboratory of Functional Materials and Devices for Special Environmental Conditions, Xinjiang Key Laboratory of Functional Crystal Materials, Xinjiang Technical Institute of Physics and Chemistry, Chinese Academy of Sciences, China; Center of Materials Science and Optoelectronics Engineering, University of Chinese Academy of Sciences, China; Research Center for Crystal Materials, CAS Key Laboratory of Functional Materials and Devices for Special Environmental Conditions, Xinjiang Key Laboratory of Functional Crystal Materials, Xinjiang Technical Institute of Physics and Chemistry, Chinese Academy of Sciences, China; Center of Materials Science and Optoelectronics Engineering, University of Chinese Academy of Sciences, China; Research Center for Crystal Materials, CAS Key Laboratory of Functional Materials and Devices for Special Environmental Conditions, Xinjiang Key Laboratory of Functional Crystal Materials, Xinjiang Technical Institute of Physics and Chemistry, Chinese Academy of Sciences, China; Center of Materials Science and Optoelectronics Engineering, University of Chinese Academy of Sciences, China; Research Center for Crystal Materials, CAS Key Laboratory of Functional Materials and Devices for Special Environmental Conditions, Xinjiang Key Laboratory of Functional Crystal Materials, Xinjiang Technical Institute of Physics and Chemistry, Chinese Academy of Sciences, China; Center of Materials Science and Optoelectronics Engineering, University of Chinese Academy of Sciences, China; Research Center for Crystal Materials, CAS Key Laboratory of Functional Materials and Devices for Special Environmental Conditions, Xinjiang Key Laboratory of Functional Crystal Materials, Xinjiang Technical Institute of Physics and Chemistry, Chinese Academy of Sciences, China; Center of Materials Science and Optoelectronics Engineering, University of Chinese Academy of Sciences, China

## Abstract

A fluorination modulation theory embeds nonmetalfluorine covalent bonds into tetrahedral units, breaking local symmetry and stabilizing electronic structures to enable rational design of high-performance vacuum ultraviolet nonlinear optical crystals.

Vacuum-ultraviolet (VUV) nonlinear optical (NLO) crystals are essential for generating coherent light at wavelengths below 200 nm, underpinning applications in advanced lithography, ultrafast photonics, and high-resolution spectroscopy [1–5]. In particular, efficient VUV frequency conversion is essential for enabling direct access to the ultra-narrow ^229m^Th nuclear clock transition, which provides a direct frequency bridge between nuclear and atomic clocks [1]. Despite decades of intensive research, practical VUV NLO materials remain exceedingly rare. Their discovery is fundamentally constrained by a set of exceptionally stringent and strongly coupled requirements: large second-order nonlinearity, sufficient birefringence for short-wavelength phase matching, and intrinsic VUV transparency. This ‘three-fold challenge’ constitutes the central bottleneck in VUV NLO materials research [6,7].

To address this bottleneck, conventional theoretical frameworks for NLO materials have largely focused on the microscopic origin of second-order polarization, successfully guiding the development of borate-based systems dominated by planar π-conjugated units (e.g. [BO_3_]) [8]. From a multiscale perspective, macroscopic second-order nonlinear response arises from the coherent superposition of microscopic hyperpolarizabilities and their orientational ordering, while birefringence depends jointly on local polarizability anisotropy and long-range framework topology. Notably, tetrahedron-based systems inherently possess wide bandgaps and high chemical stability, which are favorable for VUV transparency. However, their nearly isotropic local symmetry makes it extremely challenging to enhance optical anisotropy, which severely restricts their application in VUV NLO materials. Consequently, tetrahedral anionic units have long been regarded as unfavorable for VUV nonlinear optics, not due to electronic limitations, but because of the absence of an effective mechanism to break local symmetry and coherently amplify anisotropy across the lattice. This constraint has fundamentally hindered the rational exploration of tetrahedron-based VUV NLO materials.

Here, we establish a fluorination modulation theory for designing VUV NLO crystals that elevates fluorination from a compositional modification to an active symmetry-control paradigm. By introducing nonmetal–fluorine (*A*–F) covalent bonding into tetrahedral anionic units, fluorination serves as a controllable handle to break local symmetry, reshape anionic connectivity, and stabilize band-edge electronic structures in a correlated manner. This approach enables cooperative modulation of three key macroscopic properties: second-order NLO coefficient (*d*), birefringence (Δ*n*), and VUV transparency (bandgap, *E*_g_), within a well-defined fluorination window, rather than through mutual trade-offs. Within this framework, tetrahedral units are transformed from passive wide-bandgap hosts into symmetry-broken, anisotropic nonlinear building blocks, providing a unified theoretical basis for overcoming long-standing limitations in VUV NLO materials design.

***Covalent nonmetal-fembedding as an active design knob.*** Conventional fluorine incorporation in optical materials has largely been treated as a structural or chemical modification, in which fluorine acts as a passive ligand coordinated to metal cations. Such metal–fluorine coordination can influence lattice packing or local crystal fields, but it does not directly address the core challenge in tetrahedron-based VUV NLO systems: activating local symmetry breaking and coherently amplifying anisotropy across the anionic framework. In contrast, covalent nonmetal–fluorine embedding introduces fluorine directly into tetrahedral anionic units, forming mixed-anion groups of the type [*A*O_4−_*_x_*F*_x_*] (e.g. *A* = B, *x* = 1, 2, 3; *A* = P, *x* = 1, 2; *A* = S, *x* = 1). In this configuration, fluorine becomes an integral component of the anionic framework rather than a peripheral modifier. Substitution of oxygen by fluorine lowers the local symmetry of tetrahedral units, induces directional hybridization, and redistributes electron density within the anion. These effects collectively enhance microscopic hyperpolarizability and polarizability anisotropy without relying on band-edge dispersion. At the same time, covalent fluorination systematically alters anionic connectivity and polymerization pathways, enabling coupled modulation of topology, orientational ordering, and electronic structure. By functioning simultaneously as a local symmetry breaker, a connectivity regulator, and a band-edge stabilizer, covalent nonmetal–fluorine embedding acts as an active design knob for tetrahedron-based VUV NLO materials. This mechanism transforms fluorination from a passive compositional adjustment into a controllable, multiscale tool for activating and organizing NLO functionality in wide-bandgap systems.

Second-order NLO responses require the absence of inversion symmetry at the microscopic level, making local symmetry breaking a prerequisite for activating hyperpolarizable units. In tetrahedral anionic groups such as [*A*O_4_], the high local symmetry leads to nearly isotropic charge distributions and intrinsically weak microscopic nonlinear responses. Covalent fluorination directly addresses this limitation. Partial substitution of oxygen by fluorine transforms symmetric [*A*O_4_] units into lower-symmetry mixed-anion groups [*A*O_4−_*_x_*F*_x_*], reducing the local point symmetry (e.g. from *T*_d_ to *C*_3v_ or *C*_2v_) and inducing pronounced electronic reorganization. Fluorine’s high electronegativity causes unequal hybridization, concentrating electron density along the *A*–F bond and altering *A*–O bonds. This creates a directional charge bias that enhances microscopic hyperpolarizability (*β*) and polarizability anisotropy (Δ*α*), strengthening the macroscopic second-order coefficient (*d*). Basic building units in fluorooxoborates [9–13] with highest occupied molecular orbital (HOMO) and lowest unoccupied molecular orbital (LUMO) distributions are shown in Figs S1 and S2, contrasting *π*-conjugated *sp*² or *sp* units with *sp*³ tetrahedral units, including fluorinated [BO_4−_*_x_*F*_x_*] groups. The B–F bond in [BO_3_F] exhibits a strong *p*-character along the B–F direction, enhancing local polarization and charge redistribution under electric fields. Fluorination thus provides a robust basis for activating NLO sources in fluorooxoborates.

***Shear-polymerization-orientation coupling.*** Macroscopic performance depends not only on unit-level enhancements but also on the spatial organization and density of polar units in the lattice. Covalent fluorination enables this through a coupled shear–polymerization–orientation (S–P–O) mechanism (Fig. 1a). Structural shear arises from fluorine-induced connectivity compression and topology reduction. Terminal F substitution in tetrahedral units induces a shear-like compression by replacing bridging oxygens, reducing available polymerization directions, and biasing structural evolution toward low-dimensional motifs, thereby suppressing excessive crosslinking and isotropic packing, beneficial to structural anisotropy. Statistical analysis of reported fluorooxoborate structures shows a dimensional shift: 93.5% (116/124, Tables S1–S3) of structures are low-dimensional (0D–2D), with ∼37.9% being 2D (47/124), compared to only ∼10% in anhydrous borates [14]. This confirms that F-induced compression plays a key role in topology control and driven dimensionality reduction. Polymerization ensures both the assembly of functional units and the continuity of the anionic framework. Fluorooxoborates retain structural connectivity via bridging oxygens, which, together with the shear-induced confinement of polymerization directions, enable the formation of low-dimensional yet extended scaffolds. This robust polymerization prevents structural fragmentation and facilitates the transmission of orientational correlations, which are essential for macroscopic optical responses. Orientation is further stabilized by terminal F groups, which lock microscopic asymmetric units in place through electrostatic interactions, thereby reducing orientational disorder and promoting consistent alignment. This effect enhances both optical anisotropy and nonlinear polarization, leading to improved phase-matching ability and frequency-conversion performance in the VUV region. This coupled S–P–O mechanism therefore provides a structural basis for resolving the long-standing trilemma among nonlinearity, birefringence, and VUV transparency in tetrahedron-based NLO materials.

**Figure 1.**
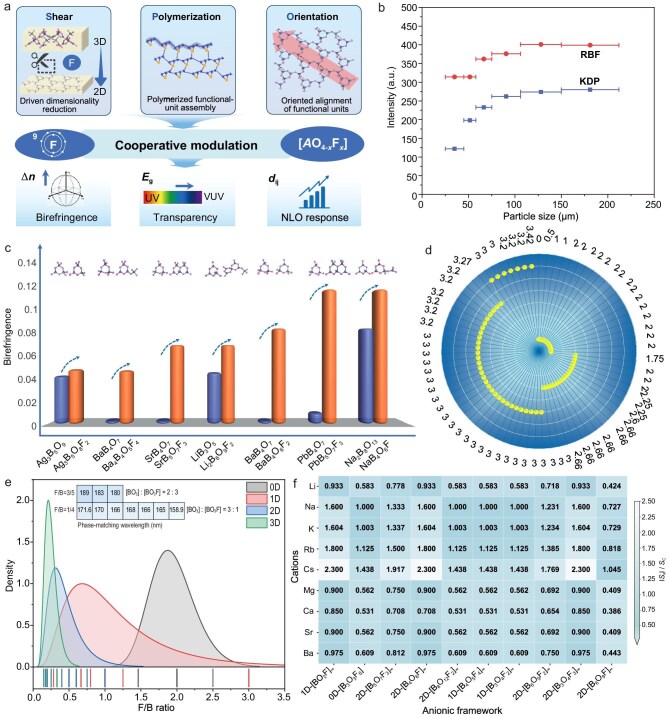
Fluorination modulation in VUV NLO crystals. (a) Schematic illustration of fluorination-enabled cooperation modulation. (b) Experimental verification using RbB_4_O_6_F (RBF). (c) Topology- and orientational-ordering-driven enhancement of birefringence from borates to fluorooxoborates within an appropriate fluorination window. Corresponding data and evolutionary pathways are summarized in Table S5. (d) Average bridging connectivity 〈*b*〉 in fluorooxoborates. (e) Distribution of F/B ratios and anionic framework dimensionality. The inset shows the typical phase-matching wavelengths for [BO_3_]/[BO_4−_*_x_*F*_x_*] ratios. (f) Heatmap of cation–anion bonding strength matching.

Guided by this mechanism, fluorooxoborates can be driven toward ordered, 2D-dominant frameworks by tuning the [BO_4−_*_x_*F*_x_*]/[BO_3_] group ratio (Fig. 1a). Two representative 2D frameworks, [B_4_O_6_F]-containing [B_4_O_8_F] ([BO_3_F] : [BO_3_] = 1 : 3) and [B_5_O_7_F_3_]-containing [B_5_O_9_F_3_] ([BO_3_F] : [BO_3_] = 3 : 2), promote anisotropic charge density distribution, structural ordering, and orientation (Figs S3–S5), beneficial for optimizing the cooperative balance among bandgap, birefringence, and second-order nonlinearity. Consistently, most reported VUV phase-matching fluorooxoborates feature 2D anionic frameworks (Table S4). These results validate group-ratio-controlled, 2D-dominant design as an effective strategy for rational VUV NLO optimization.

***Cooperative modulation mechanism of optical properties.*** Covalent fluorination enables the cooperative optimization of microscopic nonlinear polarization, birefringence, and VUV transparency in fluorinated NLO materials. In these systems, a strong second harmonic generation (SHG) response requires both a strong microscopic polarization source and effective long-range structural ordering. Enhanced microscopic polarization sources, when embedded within low-dimensional and orientationally ordered frameworks, can translate efficiently into large macroscopic SHG coefficients. This ordering stabilizes the orientational alignment of asymmetric units and increases polarization, leading to enhanced SHG responses. Representative fluorinated compounds exhibit SHG responses on the order of or exceeding those of benchmark materials such as KH_2_PO_4_ (KDP) [9–13]. A mechanism-driven reassessment of RbB_4_O_6_F further illustrates this principle: although previously reported to exhibit a powder SHG response of ∼0.8 × KDP [12], recalibrated measurements yield values of 1.4–1.5 × KDP (Fig. 1b), consistent with expectations based on source enhancement combined with order amplification.

Birefringence (Δ*n*), essential for phase matching in VUV applications, can be enhanced without excessive reliance on band-edge effects. Fluorination drives selective transformations of isotropic tetrahedral units into mixed motifs such as {[BO_3_] + [BO_3_F] or/and [BO_2_F_2_]}, which increases local polarizability anisotropy. This structural reconstruction promotes low-dimensional frameworks with stabilized orientational ordering, allowing a controlled increase in Δ*n* without excessive reliance on band-edge dispersion. For example, fluorination of Na_2_B_8_O_13_ to Na_2_B_8_O_12_F_2_ (i.e. NaB_4_O_6_F) leads to a 41.2% increase in Δ*n* (Fig. 1c). In the BaB_4_O_7_ system, fluorination produces BaB_4_O_5_F_4_ and BaB_4_O_6_F_2_, with Δ*n* increasing from 0.026 to 0.047 and 0.085, respectively. Similarly, fluorination of LiB_3_O_5_ to form Li_2_B_6_O_9_F_2_ enhances Δ*n* and shifts the phase-matching wavelength from 277 nm to 192 nm, extending into the VUV region [10]. These results demonstrate that fluorination provides a direct structural route for anisotropy control and phase-matching optimization in tetrahedron-based VUV NLO materials.

Fluorination also plays a critical role in maintaining VUV transparency by stabilizing the valence band and increasing the bandgap. Strong *A*–F bonding stabilizes anion-derived states and lowers the valence-band maximum, effectively widening the bandgap. More importantly, covalent fluorination reconstructs band-edge orbital composition by suppressing nonbonding oxygen states that would otherwise introduce localized levels near the valence edge. Electronic-structure analysis of fluorooxoborates reveals purified band edges dominated by bonding/antibonding states as well as nonbonding oxygen orbitals (Fig. S6). This band-edge purification suppresses weakly allowed low-energy transitions, thereby pushing the absorption cutoff further into the short-wavelength VUV region. In light-cation systems where nonbonding oxygen states are largely eliminated and oxygen is predominantly present as bridging oxygen, absorption edges below 150 nm can be achieved.

***Operational criteria: the effective fluorination window.*** Fluorination improves VUV NLO performance within an effective fluorination window, rather than simply increasing with fluorine content. To quantify the balance between dimensional reduction and framework continuity, we introduce the average bridging connectivity 〈*b*〉 as a key operational descriptor of anionic framework connectivity:


\begin{eqnarray*}
\left\langle b \right\rangle = \frac{1}{{{N}_A}}\mathop \sum \limits_{i = 1}^{{N}_A} {b}_i,
\end{eqnarray*}

where *b*_i_ counts the number of *A*–O–*A* bridges associated with the *i*th central atom, *N*_A_ is the number of *A* in the unit cell. In nonfluorinated systems, high 〈*b*〉 favors fully percolated 3D networks (e.g. fully bridged [BO_4_] frameworks 〈*b*〉 = 4), whereas lower 〈*b*〉 can be associated with reduced dimensionality and an increased risk of framework fragmentation (e.g. noncondensed B–O frameworks without bridging O, 〈*b*〉 = 0).

In fluorinated systems, substitution of terminal F for bridging O systematically lowers 〈*b*〉 by compressing network connectivity. This reduction, however, is nonlinear. Crucially, within a moderate fluorination region, mixed motifs composed of [BO_3_] and [BO_4−_*_x_*F*_x_*] are preferentially generated, allowing fluorinated and nonfluorinated units to assemble cooperatively while retaining a sufficient population of bridging oxygens. This coexistence preserves framework percolation and enables collective orientational ordering, rather than simple dimensional collapse. Based on the inferred relationship between the relative proportions of [BO_3_] and [BO_4−_*_x_*F*_x_*] units and the requirement for oxygen-mediated framework connectivity, we analyzed reported fluorooxoborate structures to identify the connectivity range that supports this cooperative behavior. The result converges on a critical interval of 〈*b*〉 ≈ 2.0–3.0 (for systems composed of [BO_3_] and [BO_3_F] or [BO_2_F_2_], details in Table S6), as a first effective fluorination window (Fig. 1d). Outside this range, highly crosslinked and nearly isotropic 3D networks dominate at higher 〈*b*〉, whereas excessive connectivity loss at lower 〈*b*〉 may lead to framework fragmentation and even 0D anionic framework structures. Within this region, a second effective fluorination window emerges that is governed by the F/*A* ratio (in borates, more precisely the relative proportions of [BO_3_] and [BO_4−_*_x_*F*_x_*]), defining the compositional range that stabilizes low-dimensional, orientationally ordered frameworks favorable for VUV phase matching. Figure 1e shows the relationship between the fluorination level (expressed as F/B) and structural dimensionality among known compounds. Within the 2D structural region, we further refine the optimal composition by considering experimentally reported fluorooxoborate structures. This analysis highlights the key role of the [BO_3_]/[BO_4−_*_x_*F*_x_*] ratio in tuning the phase-matching wavelength.

Within the effective fluorination window, fluorine-induced connectivity compression favors low-dimensional anionic frameworks while preserving oxygen-mediated framework continuity. This balance stabilizes orientational order and enhances structural coherence. As a result, dimensional reduction, polymerization-driven connectivity, and fluorine-induced ordering act synergistically to promote VUV NLO performance. Consistent with this picture, fluorooxoborates exhibit a higher probability of satisfying VUV phase-matching conditions than conventional borates. The average bridging connectivity 〈*b*〉, together with control of the [BO_3_]/[BO_4−_*_x_*F*_x_*] ratio (or more generally, the F/*A* ratio), therefore provides a quantitative guideline for designing tetrahedron-based VUV NLO materials.

At the microscopic level, the same fluorination range is governed by bond-valence constraints. According to the bond-valence model [15], increasing F/O substitution progressively weakens the overall bonding strength of fluorinated anionic groups (|*S*_a_|) in an approximately linear manner (Fig. S7): [BO_3_F] (0.364 valence units, vu) > [BO_2_F_2_] (0.300 vu) > [BOF_3_] (0.222 vu) > [BF_4_] (0.125 vu). On this basis, we establish a valence-matching principle for fluorooxoborates, whereby structural stability is maximized when the anionic bonding strength |*S*_a_| matches the cation bonding strength *S*_c_ (|*S*_a_| ≈ *S*_c_). Figure 1f maps representative alkali and alkaline-earth cations against typical fluorinated anionic frameworks, demonstrating that appropriate cation selection can selectively stabilize targeted anionic structures. This cation–anion matching provides a bond-level origin for the effective fluorination window and the associated framework connectivity.

***Design rules, transferability, and boundaries.*** This fluorination-enabled co-design framework yields three transferable design rules: (i) prioritizing covalent *A*–F incorporation within tetrahedral anions rather than relying solely on highly coordinated metal–F interactions; (ii) tuning fluorine content, near the connectivity threshold to maintain a wide bandgap while balancing dimensional reduction with framework continuity, while further adjusting the F/*A* ratio (e.g. the relative proportion of [BO_3_] to [BO_4−_*_x_*F*_x_*] units in borates) to optimize optical anisotropy and SHG; and (iii) promoting orientational order through terminal-fluorine interactions and appropriate cation-field effects to enable coherent amplification of local responses. Although demonstrated primarily in borate systems, this mechanism is largely chemistry-agnostic and is extendable to other tetrahedral anion frameworks, such as phosphates and sulfates. Nevertheless, differences in electronic structure and anion polymerization behavior may shift the optimal fluorination window across chemistries. For example, the electron-deficient nature of boron enables [BO_4−_*_x_*F*_x_*] to induce framework polymerization through bridging oxygens, whereas phosphate and sulfate frameworks show weaker tendencies toward anion polymerization. While this work highlights nonmetal–F embedding as the primary driver for activating anisotropy and nonlinear response, the fluorination modulation theory is inherently broader. In particular, low-coordination metal–F interactions (e.g. Li, Be), although not the focus here, can play an important complementary role in stabilizing band edges and improving VUV transparency, and therefore also fall within the general fluorination-based modulation framework.

Taken together, fluorination modulation theory establishes a design framework for the design of VUV NLO crystals, moving beyond empirical trial-and-error toward mechanism-guided materials design. By identifying covalent nonmetal–F embedding and defining an effective fluorination window, it enables cooperative modulation of polarization, topology, orientational order, and band-edge stability, helping overcome trade-offs between NLO response, birefringence, and VUV transparency. Its validation by the realization of the ABF family and state-of-the-art VUV frequency-conversion performance further demonstrates the practical significance of this theory beyond conceptual design. This framework thus defines a new performance benchmark for VUV NLO crystals, where symmetry-broken tetrahedral units, orientational coherence, and wide bandgaps can be simultaneously achieved. At the same time, the theory framework remains at an early stage, and further exploration on chemical diversity, structural evolution, and structure–property relationships is required. By integrating theory-guided structure generation, orientational-order engineering, and multi-objective optimization, this framework provides a pathway toward next-generation VUV NLO materials with performance beyond the current state of the art.

## Supplementary Material

nwag259_Supplemental_File
