# Supplementary material for: Fluorination modulation theory for vacuum-ultraviolet nonlinear optical crystals (I): foundation and framework
Source: Natl Sci Rev. 2026 May 18;13(14):nwag259. doi: 10.1093/nsr/nwag259 (PMC13411276; doi:10.1093/nsr/nwag259)
Supplement: nwag259_Supplemental_File [file nwag259_supplemental_file.pdf]

Supporting Information

Fluorination modulation theory framework for vacuum-ultraviolet nonlinear optical crystals (I): foundation and framework

Zhihua Yang[a,b], Abudukadi Tudi[a,b], Min Zhang[a,b], Miriding Mutailipu[a,b], Fangfang Zhang[a,b], Shilie Pan[a,b]*

[a] Research Center for Crystal Materials; CAS Key Laboratory of Functional Materials and Devices for Special Environmental Conditions; Xinjiang Key Laboratory of Functional Crystal Materials; Xinjiang Technical Institute of Physics and Chemistry, Chinese Academy of Sciences, 40-1 South Beijing Road, Urumqi 830011, China

[b] Center of Materials Science and Optoelectronics Engineering, University of Chinese Academy of Sciences, Beijing 100049, China

* Correspondence, E-mail: slpan@ms.xjb.ac.cn

Table of Contents

Title	Page
**Experimental Procedures**	
**Table S1.** Typical fluorooxoborates without hydroxyl group OH, the dimension distribution of anionic framework and the bridging connectivity.	S4
**Table S2.** Typical fluorooxoborates with hydroxyl group OH.	S7
**Table S3.** Typical fluorooxoborates (general raw materials do not contain, e.g. $LiBF_4$).	S8
**Table S4.** Typical fluorooxoborates with VUV phase-matching wavelength.	S9
**Table S5.** Comparison of birefringence.	S10
**Table S6.** Analysis of average bridging connectivity <*b*> fluorooxoborates only containing $[BO_3] + [BO_{4-x}F_x]$ and all O are 2-coordinated bridging oxygens.	S11
**Figure S1.** Typical B-O/F group and the HOMO, LUMO and energy gap.	S12
**Figure S2.** Electron density for $NH_4B_4O_6F$ (a) and $SrB_5O_7F_3$(b), respectively.	S13
**Figure S3.** Anisotropic charge density distribution and electron localization function of $[B_4O_8F]$ and $[B_5O_9F_3]$.	S14
**Figure S4.** Electron density for $NH_4B_4O_6F$ (a) and $SrB_5O_7F_3$ (b), respectively.	S15
**Figure S5.** Electronic location function of $[B_4O_8F]$ in CsB_4O_6F.	S16
**Figure S6.** Optical absorption density distribution in a fluorooxoborate ($NH_4B_4O_6F$, ABF), proving fluorination purifies band-edge states.	S17
**Figure S7.** Anion bonding strength for fluorinated units.	S18

Experimental Procedures

Synthesis and Crystal Growth.

All the reagents including $RbBF_4$ (Aladdin, 99%), H_3BO_3 (Aladdin, 99.5%) are analytical grade from commercial sources without further purification. The title compound was obtained conventional solid-state reactions. The loaded compositions are: $RbBF_4$, H_3BO_3 with a molar ratio of 1:4. Single crystals of the title compound were grown by spontaneous crystallization using $LiBF_4$ as the flux. The compositions of respective mixtures are as follows: RbF (0.104 g, 1 mmol), $LiBF_4$ (0.094 g, 1 mmol), and B_2O_3 (0.139 g, 2 mmol) for RBF. The mixtures were thoroughly ground, placed in separate platinum crucibles, and gradually heated to 400 °C and held at this temperature in air for 6h. The temperature was slowly cooled down to 200°C at a rate of $1°C\cdot h^{-1}$ before turned off the furnace. The sub-millimeter-sized crystals were separated mechanically from the crucible.

Second-Harmonic Generation Measurements. Powder SHG measurements of the title compound was operated by the Kurtz−Perry method [1] using a Q-switched Nd:YVO_4 solid-state laser at 1064 nm for visible and UV SHG, respectively. Polycrystalline powders of RbB_4O_6F were ground and divided into the different particle size ranges: 38−55, 55−88, 88−105, 105−150, 150-200 and 200−250 μm. The sample was loaded in 1 mm thick aluminum holders containing an 8 mm diameter hole, pressed between glass microscope cover slides, and secured. Microcrystallines of KDP were also sieved into the same particle size range and used as the references, respectively.

Numerical Calculation Details. The electronic structure and the optical property of the title compound were calculated on the basis of *ab initio* calculations implemented in the CASTEP package through density functional theory (DFT).[2,3] During the calculation, the generalized gradient approximation (GGA) with Perdew-Burke-Ernzerhof (PBE) functional was adopted to calculate the exchange-correlation potential.[4,5] In addition, Heyd–Scuseria–Ernzerhof (HSE06) hybrid functional implemented in PWmat code was adopted to obtain more accurate bandgaps.[6] The electron configuration set was adopted with the norm-conserving pseudopotential (NCP. The numerical integration of the Brillouin zone adopted the default "fine" settings. [7] The energy range of the band gap and density of states is selected as 20 eV, and the optical property for the typical fluorooxoborates [8-15] is 60 eV. The linear optical properties of the title compounds were examined based on the dielectric function $\varepsilon(\omega) = \varepsilon_1(\omega) + i\varepsilon_2(\omega)$. The imaginary part of dielectric function, $\varepsilon_2(\omega)$, can be calculated from the electronic transition between the occupied and the unoccupied states by the following formula:

$$\varepsilon_2(\omega) = \frac{4\pi\ell^2}{\Omega}\lim_{q\to 0}\frac{1}{q^2}\times\sum\nolimits_{c,v,k} 2\omega_k\delta(E_c - E_v - \omega)\,|\langle c\,|\,e\cdot q\,|\,v\rangle|^2$$

where Ω is the volume of the elementary cell, v and c depict the valence bands (VBs) and the conduction bands (CBs), respectively, ω is the frequency of the incident light, the $\langle c\,|\,e\cdot q\,|\,v\rangle$ is the integrated optical transitions from the valance states to the conduction states. The real part, $\varepsilon_1(\omega)$, is obtained by the Kramers-Kronig transformation,[16] accordingly the refractive indices and the birefringence (Δn) can be calculated. The Gaussian 09 package was employed to explore the electronic structures of B-O/F anionic groups at molecular level. Electronic localization functions for the groups were calculated using Multiwfn.[17-18]

Results and Discussion

Table S1. Typical fluorooxoborates without hydroxyl group OH, the dimension distribution of anionic framework and the bridging connectivity.

No.	Compounds	Space groups	B/F	Dimension	<*b*>
1	$Li_2B_6O_9F_2$	*Cc*	3	3D	3
2	$LiNaB_6O_9F_2$	*Pnn*2	3	3D	3
3	$Na_2BaB_{12}O_{18}F_4$	*Pbcn*	3	3D	3
4	$BaB_5O_8F\cdot xH_2O$	*Pbca*	5	3D	16/5
5	$K_3NaB_{10}O_{16}F_2$	*C*2/*c*	5	3D	16/5
6	$Ba_2B_7O_{12}F$	*C*2/*c*	7	3D	24/7
7	PbB_5O_8F	*Pbca*	5	3D	16/5
8	$Ag_2B_5O_8F$	*Pbca*	5	3D	16/5
9	LiB_6O_9F	$Pna2_1$	6	2D	3
10	$Na_2B_6O_9F_2$	$P2_1/c$	3	2D	3
11	$(NH_4)_2B_6O_9F_2$	$P2_1/c$	3	2D	3
12	$K_2B_6O_9F_2$	*Pbca*	3	2D	3
13	$NaRbB_6O_9F_2$	$P2_1/n$	3	2D	3
14	$NaKB_6O_9F_2$	$P2_1/c$	3	2D	3
15	$K_3B_6O_9F_3$	$P2_1/c$	2	2D	3
16	α-$K_3B_6O_9F_3$	*Pc*	2	2D	3
17	$K_{1.2}Rb_{1.8}B_6O_9F_3$	*Pc*	2	2D	3
18	$K_{2.9}Cs_{0.1}B_6O_9F_3$	*Pc*	2	2D	3
19	$BaB_2O_3F_2$	$P2_1$	1	2D	3
20	$SnB_2O_3F_2$	$P3_1m$	1	2D	3
21	$PbB_2O_3F_2$	$P3_1m$	1	2D	3
22	NaB_4O_6F	*C*2	4	2D	3
23	NaB_4O_6F	*C*2/*c*	4	2D	3
24	RbB_4O_6F	$Pna2_1$	4	2D	3
25	CsB_4O_6F	$Pna2_1$	4	2D	3
26	$SrB_4O_6F_2$	*P-1*	2	2D	3
27	$CaB_4O_6F_2$	*P-1*	2	2D	3
28	$BaB_4O_6F_2$	$P2_1/n$	2	2D	3
29	$CsKB_8O_{12}F_2$	*P*321	4	2D	3
30	$CsRbB_8O_{12}F_2$	*P2c*	4	2D	3

31	$BaB_8O_{12}F_2$	*R3c*	4	2D	3
32	$NH_4B_4O_6F$	*Pna*2$_1$	4	2D	3
33	$CsKB_8O_{12}F_2 \cdot CsI$	*R*32	4	2D	3
34	$CsNH_4B_8O_{12}F_2 \cdot CsI$	*R*32	4	2D	3
35	$CaB_5O_7F_3$	*Cmc*2$_1$	5/3	2D	3
36	$SrB_5O_7F_3$	*Cmc*2$_1$	5/4	2D	3
38	$PbB_5O_7F_3$	*Cmc*2$_1$	5/5	2D	3
39	$MgB_5O_7F_3$	*Cmc*2$_1$	5/6	2D	3
40	$Li_2Na_{0.9}K_{0.1}B_5O_8F_2$	*Pbca*	5/2	2D	16/5
41	$K_{0.6}Rb_{2.4}B_5O_8F_2$	*P*2$_1$	5/2	2D	16/5
42	$Rb_3B_5O_8F_2$	*P*2$_1$	5/2	2D	16/5
43	$Li_2NaB_5O_8F_2$	*Pbca*	5/2	2D	16/5
44	$Li_2KB_5O_8F_2$	*Pbca*	5/2	2D	16/5
45	$Ba_3B_{10}O_{17}F_2 \cdot 0.1KF$	*P-1*	5	2D	34/10
46	$K_5B_{11}O_{18}F_2$	*P-1*	11/2	2D	36/11
47	$K_6B_{12}O_{19}F_4$	*Pnma*	3/1	2D	/
48	$Ag_3B_5O_8F_2$	*P 21/n*	5/2	2D	16/5
49	$K_x(NH_4)_{2-x}B_8O_{12}F_2$	*Pna*2$_1$	4	2D	3
50	$Rb_x(NH_4)_{2-x}B_8O_{12}F_2$	*Pna*2$_1$	4	2D	3
51	$CsNH_4B_8O_{12}F_2$	*Pna*2$_1$	4	2D	3
52	$KNiB_4O_6F_3$	*P*2$_1$*/c*	4/3	2D	3
53	$KCoB_4O_6F_3$	*P*2$_1$*/c*	4/3	2D	3
54	$KFeB_4O_6F_3$	*P*2$_1$*/c*	4/3	2D	3
55	BiB_2O_4F	*P*3$_2$	2	1D	(2+4)/2
56	SbB_2O_4F	*P-1*	2	1D	(2+2)/2
57	$Na_3B_7O_{11}F_2$	*Pnma*	3.5	1D	3
58	$Ba_2B_9O_{13}F_4 \cdot BF_4$	*P*	13/8	1D	26/10
59	$Li_2B_3O_4F_3$	*P*2$_1$2$_1$2$_1$	1	1D	8/3
60	$RbB_3O_4F_2$	*P*2$_1$*/c*	3/2	1D	8/3
61	α-$KB_3O_4F_2$	*P*2$_1$*/n*	3/2	1D	8/3
62	β-$KB_3O_4F_2$	*P*2$_1$*/n*	3/2	1D	8/3
63	$NaRbB_3O_4F_3$	*P*2$_1$*/c*	1	1D	8/3
64	$NaKB_3O_4F_3$	*Pbcn*	1	1D	8/3

65	$K_2B_3O_4F_3$	*Pbcn*	1	1D	8/3
66	$KCsB_3O_4F_3$	*Pbcn*	1	1D	8/3
67	α-$BaB_4O_5F_4$	$P2_1$	1	1D	9/4
68	β-$BaB_4O_5F_4$	$P2_1/c$	1	1D	9/4
69	β-$BaBOF_3$	$P2_1/c$	1/3	1D	2
70	α-$BaBOF_3$	$P2_1$	1/3	1D	2
72	γ-$BaBOF_3$	$P2_1/c$	1/3	1D	2
73	$Na_4B_8O_9F_{10}$	*Ama2*	4/5	1D	14/8
74	$Cs_2B_3O_4F_3$	$P2_1/c$	1	1D	8/3
75	$KNaB_3O_4F_3$	*Pbcn*	1	1D	8/3
76	$K_{10}B_{13}O_{15}F_{19}$	*R*3*m*	13/19	0D	2
77	$Rb_{10}B_{13}O_{15}F_{19}$	*R*3*m*	13/19	0D	2
78	$K_{0.42}Rb_{2.58}B_3O_3F_6$	*Pbcn*	1/2	0D	2
79	$Na_3B_3O_3F_6$	*C*2/*c*	1/2	0D	2
80	$Rb_3B_3O_3F_6$	*Pbcn*	1/2	0D	2
81	$Cs_3B_3O_3F_6$	*Pbcn*	1/2	0D	2
82	$K_2RbB_3O_3F_6$	$P2_1/c$	1/2	0D	2
83	$K_{1.66}Rb_{1.34}B_3O_3F_6$	$P2_1/c$	1/2	0D	2
84	$K_{2.64}Cs_{0.36}B_3O_3F_6$	$P2_1/c$	1/2	0D	2
85	$KCs_2B_3O_3F_6$	$P2_1/c$	1/2	0D	2
86	$Na_{0.76}Rb_{2.24}B_3O_3F_6$	$P2_1/c$	1/2	0D	2
87	$Cs_{1.29}Rb_{1.71}B_3O_3F_6$	$P2_1/c$	1/2	0D	2
88	$K_{2.3}Cs_{0.7}B_3O_3F_6$	$P2_1/c$	1/2	0D	2
89	$K_3B_3O_3F_6$	$P2_1/n$	1/2	0D	2
90	$K_3H(B_2OF_6)F_2$	*Pnma*	1/4	0D	1
91	$Rb_3H(B_2OF_6)F_2$	*Pnma*	1/4	0D	disorder
92	$(N_2H_5)_2B_2OF_6$	*Pnma*	1/3	0D	0.5
93	$(NH_4)_5(B_2OF_6)_2F$	$I4_1/a$	4/13	0D	1
94	$Cs_4B_4O_3F_{10}$	$P2_1/c$	4/10	0D	2
95	$SbBO_2F_2$	*C*2/*c*	1/2	0D	0

Table S2. Typical fluorooxoborates with hydroxyl group OH.

No.	Formula	Space group	Symmetry	Dimension
1	$Ba(B_2OF_3(OH)_2)_2$	*C*2/*m*	CS	0D
2	$NaB_3O_4F(OH)$	*P*2₁/*c*	CS	1D
3	$Na[B_3O_3F_2(OH)_2][B(OH)_3]$	*P*-1	CS	0D
4	$LiB_5O_5F_2(OH)_4$	*P*2₁/*m*	CS	0D
5	$[C(NH_2)_3][B_3O_3F_2(OH)_2]$	*P*1	NCS	0D
6	$[C(NH_2)_3]_2[B_3O_3F_4(OH)]$	*P*1	NCS	0D
7	$(NH_4)_4[B_{12}O_{16}F_4(OH)_4]$	*P*4/*ncc*	CS	0D
8	$CsB_3O_3F_2(OH)_2$	*P*2₁/*c*	CS	0D
9	$Rb_2B_6O_9F(OH)$	*Cc*	NCS	2D
10	$(NH_4)_2[B_3O_3F_4(OH)]$	*C*2/*c*	CS	0D
11	$Rb_2[B_3O_3F_4(OH)]$	*C*2/*c*	CS	0D
12	$Cs_2[B_3O_3F_4(OH)]$	*C*2/*c*	CS	0D
13	$Rb[B_3O_3F_2(OH)_2]$	*C*2/*m*	CS	0D
14	$K_2[B_2O_2F_2(OH)_2]\cdot 2H_2O$	/	/	/
15	$(NH_4)_2[B_2O_2F_2(OH)_2]\cdot 2H_2O$	/	/	/
16	$K_5[B_3O_3F_4(OH)]_2(NO_3)$	*C*2/*c*	CS	0D
17	α-$K_2B_3O_3F_4(OH)$	*C*2/*c*	CS	0D
18	β-$K_2B_3O_3F_4(OH)$	*Ama2*	NCS	0D
19	$[C_3N_6H_7]_2[B_3O_3F_4(OH)]$	*P*-1	CS	0D
20	$(NH_4)[C(NH_2)_3][B_3O_3F_4(OH)]$	*C*2/*c*	CS	0D
21	$K_8Cs_2B_{15}O_{14}(OH)_7F_{20}\cdot H_2O$	*P*2₁/*c*	CS	0D
22	β-$Rb_2B_3O_3F_4(OH)$	*Ama*2	NCS	0D
23	$Rb_2B_3O_3F_4(OH)$	*C*2/*c*	CS	/
24	$(C_3N_2H_5)B_3O_3F_2(OH)_2$	*P-1*	CS	0D
25	$[Ph_4P]B_3O_3F_2(OH)_2$	*C*2/*m*	CS	0D
26	$[Ph_3MeP]B_3O_3F_2(OH)_2$	*C*2/*m*	CS	0D

27	$Cs(NH_4)_3[B_3O_3F_4(OH)]_2$	*C*2/*c*	CS	0D
28	$RbCs_5[B_3O_3F_4(OH)]_3$	*Ama*2	NCS	0D
29	$[CN_4H_7]_2[B_3O_3F_4(OH)]$	*P*2$_1$/*c*	CS	0D

Table S3. Typical fluoroborates (general raw materials do not contain, e.g. $LiBF_4$).

No.	Formula	Space group	Symmetry	Dimension
1	$[C_3N_6H_7][BF_4]\cdot H_2O$	*P*-1	CS	0D
2	$[C(NH_2)_3][BF_4]$	*R*3*m*	NCS	0D

Table S4. Typical fluorooxoborates with VUV phase-matching wavelength.

	Compounds	VUV phase-matching wavelength (nm)
1	$NH_4B_4O_6F$	158
2	NaB_4O_6F	166
3	RbB_4O_6F	165
4	CsB_4O_6F	171.6
5	$CsKB_8O_{12}F_2$	170
6	$CsRbB_8O_{12}F_2$	<200
7	$CaB_5O_7F_3$	183
8	$SrB_5O_7F_3$	180
9	$MgB_5O_7F_3$	189
10	$Li_2B_6O_9F_2$	192
11	$Rb_{1.34}(NH_4)_{0.66}B_8O_{12}F_2$	161.5
12	$Rb_{0.5}(NH_4)_{1.5}B_8O_{12}F_2$	166.4
13	$CsNH_4B_8O_{12}F_2$	167.8
14	$[C(NH_2)_3][B_3O_3F_2(OH)_2]$	190
15	$[C(NH_2)_3]_2[B_3O_3F_4(OH)]$	195
16	$C(NH_2)_3BF_4$	193.2

Table S5. Comparison of birefringence.

Ag	$Ag_3B_5O_9$	$Ag_3B_5O_8F_2$
	0.042	0.048
Ba	BaB_4O_7	$BaB_4O_5F_4$
	0.0026	0.047
Ba	BaB_4O_7	$SrB_5O_7F_3$
	0.0026	0.07
Li	LiB_3O_5	$Li_2B_6O_9F_2$
	0.045	0.07
Ba	BaB_4O_7	$BaB_4O_6F_2$
	0.0026	0.085
Pb	PbB_4O_7	$PbB_5O_7F_3$
	0.0091	0.12
Na	$Na_2B_8O_{13}$	NaB_4O_6F
	0.085	0.12

* Evolutionary path: $Ag_3B_5O_9 \rightarrow Ag_3B_5O_8F_2$, $BaB_4O_7 \rightarrow BaB_4O_5F_4$, $BaB_4O_7 \rightarrow SrB_5O_7F_3$, $LiB_3O_5 \rightarrow Li_2B_6O_9F_2$, $BaB_4O_7 \rightarrow BaB_4O_6F_2$, $PbB_4O_7 \rightarrow PbB_5O_7F_3$ (PbB_4O_7+BF_3), $Na_2B_8O_{13} \rightarrow NaB_4O_6F$ ($Na_2B_8O_{12}F_2$).

Table S6 Analysis of average bridging connectivity <*b*> fluorooxoborates only containing [BO_3] + [$BO_{4\text{-}x}F_x$] and all O are 2-coordinated bridging oxygens.

Structural Motif combination	Structural condition (O all as 2-coordinated bridging oxygens)	<*b*>	Structural features
[BO_3] + [BO_3F]	[BO_3] : [BO_3F] = any ratio	3	A 3D network tends to form when [BO_3F] is abundant; thus, an appropriate ratio is required to ensure the synergy of the three key properties.
[BO_3] + [BO_2F_2]	[BO_3] : [BO_2F_2]→∞	3	An appropriate ratio is required to ensure the synergy of the three key properties.
	[BO_2F_2] : [BO_3]→∞	2	
[BO_3] + [BOF_3]	[BO_3] : [BOF_3]→∞ (rarely if O all are 2-coordinated bridging oxygens)	3	Such structures tend to favor 0D clusters and 1D chains. Systematic studies will be carried out in future work due to the limited number of reported structures to date.
	[BOF_3] : [BO_3]→∞ (rarely observed due to terminal F effect)	1	

* The above analysis only covers derivations for fluorooxoborates containing solely the aforementioned group combinations. It does not take into account other more complex cases involving various mixed groups, such as [BO_4].

* Many of these extreme cases are unlikely to be realized in practice and are included here mainly to illustrate the possible range of ⟨*b*⟩.

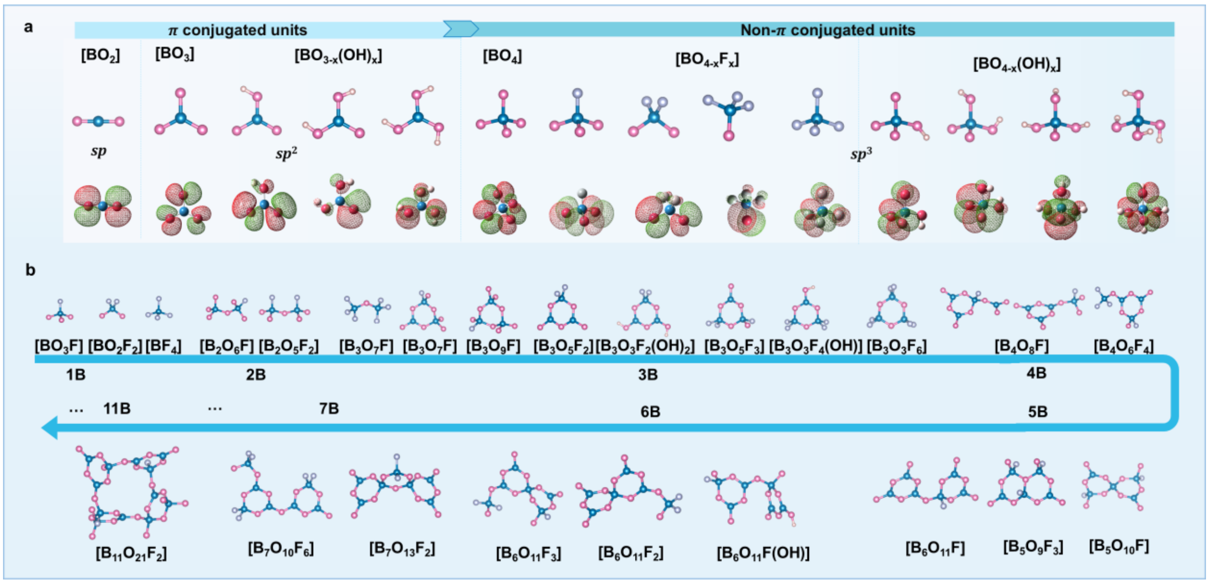

Figure S1. Represented units in fluorooxoborates, where covalent nonmetal–F embedding in tetrahedral anionic units enables unequal hybridization (a). Fundamental structural units (b).

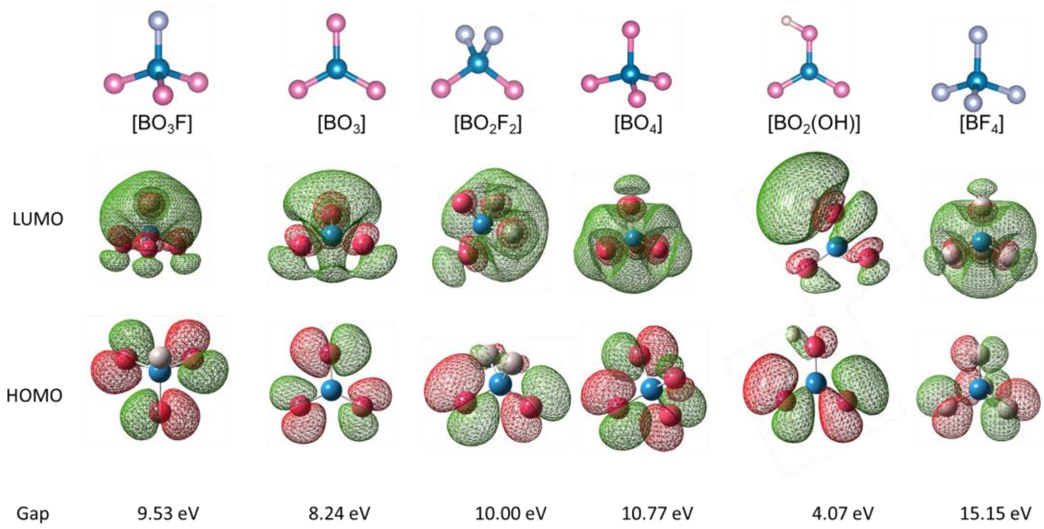

Figure S2. Typical B-O/F group and the HOMO, LUMO and energy gap.

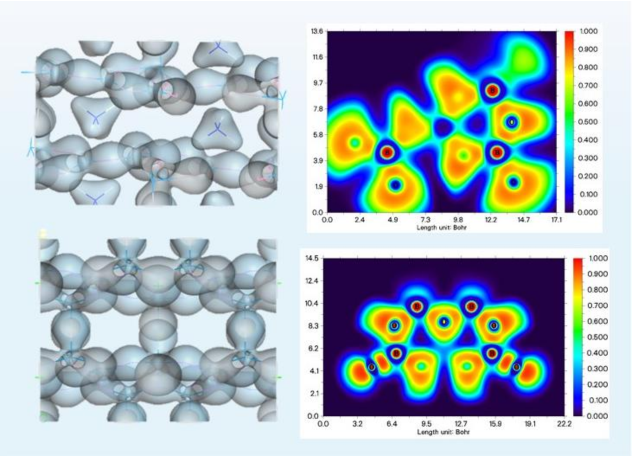

Figure S3. Anisotropic charge density distribution and electron localization function of [B_4O_8F] and [$B_5O_9F_3$].

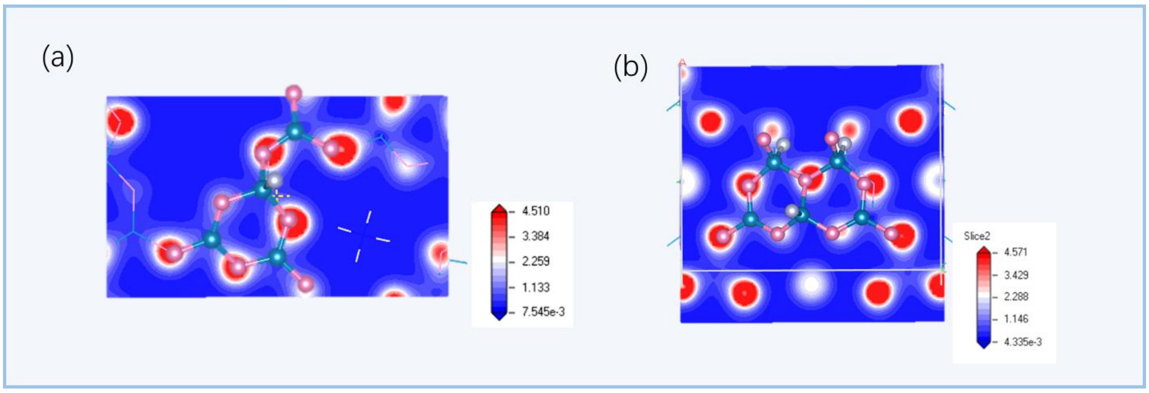

Figure S4. Electron density for $NH_4B_4O_6F$ (a) and $SrB_5O_7F_3$(b), respectively.

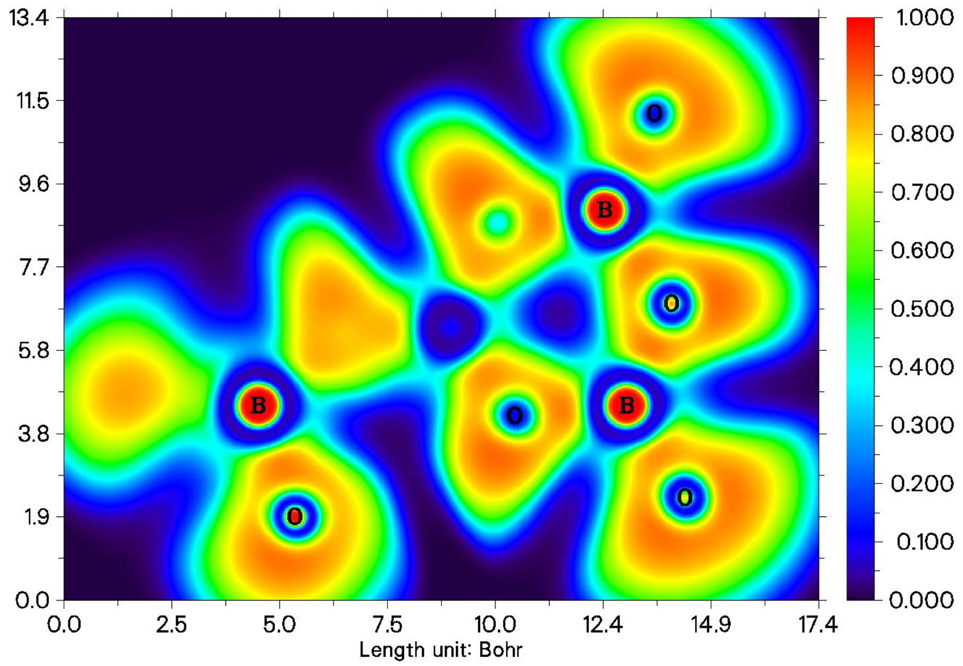

Figure S5. Electronic location function of $[B_4O_8F]$ in CsB_4O_6F.

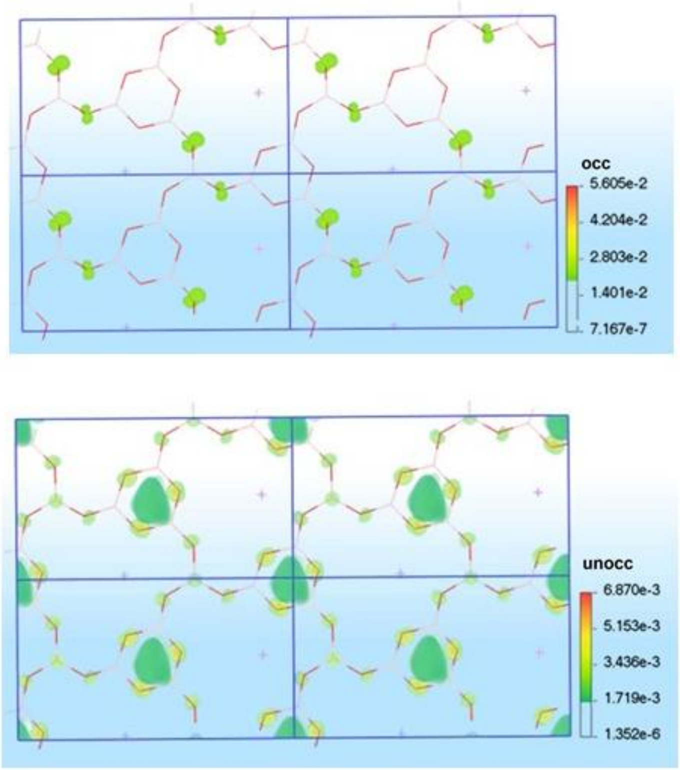

Figure S6. Optical absorption density distribution in a fluorooxoborate ($NH_4B_4O_6F$, ABF), proving fluorination purifies band-edge states.

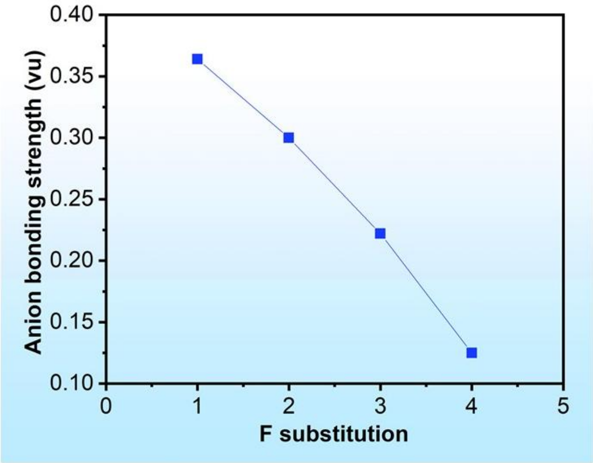

Figure S7. Anion bonding strength for fluorinated units.

References

1. S. K. Kurtz, T. T. Perry, *J. Appl. Phys.* **1968**, *39*, 3798.
2. P. Hohenberg, W. Kohn, *Phys. Rev.* **1964**, *136*, B864.
3. W. Kohn, L. Sham, *Phys. Rev.* **1965**, *140*, A1133.
4. J. P. Perdew, K. A. Jackson, M. R. Pederson, D. J. Singh, C. Fiolhais, *Phys. Rev. B* **1992**, *46*, 6671.
5. J. P. Perdew, K. Burke, M. Ernzerhof, *Phys. Rev. Lett.* **1996**, *77*, 3865.
6. B. L. Wu, C. L. Hu, F. F. Mao, R. L. Tang, J. G. Mao, *J. Am. Chem. Soc.* **2019**, *141*, 10188.
7. H. J. Monkhorst, J. D. Pack, *Phys. Rev. B* **1976**, *13*, 5188.
8. B. B. Zhang, G. Q. Shi, Z. H. Yang, F. F. Zhang, S. L. Pan, *Angew. Chem. Int. Ed.* 2017, *56*, 3916-3919.
9. G. Q. Shi, Y. Wang, F. F. Zhang, B. B. Zhang, Z. H. Yang, X. L. Hou, S.L. Pan, K. R. Poeppelmeier, *J. Am. Chem. Soc*. 2017, *139*, 10645.
10. X. F. Wang, Y. Wang, B. B. Zhang, F. F. Zhang, Z. H. Yang, S. L. Pan. *Angew. Chem. Int. Ed*. 2017, *56*, 14119.
11. G. Peng, N. Ye, Z. S. Lin, L. Kang, S. L. Pan, M. Zhang, C. S. Lin, X. F. Long, M. Luo, Y. Chen, Y. H. Tang, F. Xu, T. Yan, *Angew. Chem. Int. Ed.* 2018, *57*, 8968.
12. M. Luo, L. Fei, Y. X. Song, D. Zhao, F. Xu, N. Ye, Z. S. Lin, *J. Am. Chem. Soc*. 2018, *140*, 6509–6516.
13. Y. Wang, B. B. Zhang, Z. H. Yang, S. L. Pan. *Angew. Chem. Int. Ed*. 2018, 57, 2150.
14. M. Xia, F. M. Li, M. Mutailipu, S. J. Han, Z. H. Yang, S. Pan, *Angew. Chem., Int. Ed*. 2021, *60*, 14650.
15. M. Mutailipu, M. Zhang, B. Zhang, L. Wang, Z. Yang,X. Zhou, S. Pan, *Angew. Chem., Int. Ed.* 2018, *57*, 6095.
16. E. D. Palik, Handbook of Optical Constants of Solids; Academic Press: New York, 1985.
17. T. Lu, F. Chen, *J. Comput. Chem*. 2012, 33, 580-592.
18. T. Lu, *J. Chem. Phys*., 2024, 161, 082503 .
